# The League of Mercy

**Published:** 1903-05-30

**Authors:** 


### The Hospital.

A JOURNAL OF

The Medical Sciences and Hospital Administration.

EDITED BY SIR HENRY BURDETT, K.C.B.

VOL. XXXIV.—No. 870. [MAY 30, 1903.

### THE LEAGUE OF MERCY.

ALTHOUGH King Edward's Hospital Fund has been actively at work for five years, the public scarcely yet appreciate that the underlying motive was to secure a revenue for the hospitals, not alone from the rich but more especially by enlisting the sympathies and the support of an army of small givers. The practical interest of his Majesty the King in the Fund, and his tenacity of purpose are illustrated by his having secured a Royal Charter for the League of Mercy, the chief object of which is to set up a machinery in the metropolitan boroughs and throughout the home counties whereby every person, however small his means, may be afforded the readiest facilities to give small subscriptions of 1s. a year and upwards to the hospitals with the minimum of trouble to himself and an absolute guarantee that every penny contributed shall be devoted without deduction to the work of the hospitals. The details of this organisation show a far-reaching appreciation of the difficulties to be overcome, and the means whereby alone success can be hoped for. It is a league of personal service, and we take it that its usefulness is immeasurably greater than any good which may result from the contributions raised through its agency, however great those contributions may be. The hale owe a duty of service to the sick and suffering. Man is so constituted that, so long as he is blessed with perfect health and is able to live his life without serious illness of any kind, so long will he remain oblivious of and callous to the suffering and illness around him.

The League of Mercy aims at arousing the sympathies of the hale and hearty members of the race of both sexes. By quickening their intelligence and arousing their sympathies, it tends to humanise and soften the daily life of the people. By enlisting an army of workers, each member of which agrees to give a measure of personal service to the cause of the sick, it stiffens the fibre and raises the standard of individual citizens. Everyone is asked to devote a few hours each year to the work of the League, which consists in making the work of the hospitals known, and so each worker is enabled to secure a number of small contributions from those whom he habitually meets in daily life. The individual gifts are so small that they are inappreciable; but the total sum raised by this voluntary agency already amounts to something like £10,000 a year, and there is every reason to hope that at least £25,000 a year will be contributed in this way to the hospitals, and that even double that amount may be raised by members of the League within the next five years. Thus the King's organisation not only helps the hospitals, but it helps the people to help themselves. It has initiated a crusade of personal service, humanising in its influence, which is calculated to raise the tone and character of the individual. Although the labour involved in the initial organisation has been considerable, the progress already made affords the best guarantee, that the League of Mercy will endure and extend, so that one day it will probably be found at work in every large centre of population throughout the British Empire. The people owe this excellent League of Mercy to the foresight and judgment of their Sovereign. In view of the good it is capable of yielding to all classes, no more kingly act could a modern ruler have rendered to his subjects. It reveals the innermost mind of King Edward, and the League of Mercy may prove a living monument to the largeness of his heart as a man, and to the wisdom of his methods as a King and Emperor.

It has been the dream of many zealous friends of the hospitals, especially in London, to arouse the sympathy of the humbler citizens and to secure from them regular support for the voluntary hospitals. Will the League of Mercy succeed in doing this where other organisations have failed? We think it will. During the winter months several town halls in the metropolitan boroughs have been crowded with residents who have attended under the auspices of the League of Mercy to hear about the hospitals, and the enthusiasm manifested has been most encouraging. More important still, as the outcome of these meetings, the scheme of the League has been widely popularised.

Another excellent effect which the League has had is to unite all classes, from the highest to the lowest, in support of the hospitals. The garden party at Marlborough House on Friday the 22nd instant exhibited this fact in a striking manner. The Prince and Princess of Wales received upwards of 1,000 officers and members of the League of Mercy, and amongst them were representatives of every division of the peerage, the leading magnates of the City, representatives of county and borough life, many doctors and nurses, and practically all sections of the community, one of the members, a railway porter, attending in his uniform as proud as a prince and enjoying himself with the best. The example of their Royal Highnesses should stimulate the presidents in each parliamentary division to give each year a reception or garden party at which all classes are represented through their membership of the League of Mercy. More important still, the example of their Majesties the King and Queen and the members of the Royal family in giving personal service in the days of health has aroused general interest. A spirit of loving desire to do something for the wayfarer who on life's journey is struck down by sickness or accident now pervades all classes of Londoners, and cannot fail to prove of immense importance to the well-being of all classes, quite apart from the immediate benefits to the Hospital Fund.

